# Characterization of a novel variant in *KCNJ16* , encoding K_ir_5.1 channel

**DOI:** 10.14814/phy2.70083

**Published:** 2024-10-16

**Authors:** Biyang Xu, Vladislav Levchenko, Ruslan Bohovyk, Ameneh Ahrari, Aron M. Geurts, Valerie Sency, Baozhong Xin, Heng Wang, Alexander Staruschenko

**Affiliations:** ^1^ Department of Molecular Pharmacology and Physiology University of South Florida Tampa Florida USA; ^2^ Department of Physiology Medical College of Wisconsin Milwaukee Wisconsin USA; ^3^ DDC Clinic for Special Needs Children Middlefield Ohio USA; ^4^ Hypertension and Kidney Research Center, University of South Florida Tampa Florida USA; ^5^ James A. Haley Veterans' Hospital Tampa Florida USA

**Keywords:** *KCNJ16* mutation, K_ir_ channels, K_ir_5.1, potassium channel, salt‐sensitive hypertension

## Abstract

The essential role of the inwardly rectifying potassium channel K_ir_5.1 (*KCNJ16*) in controlling electrolyte homeostasis and blood pressure has been demonstrated in human and animal studies. Previous studies have identified several bi‐allelic mutations of *KCNJ16* in humans, causing severe hypokalemia, renal salt wasting, and disturbed acid–base homeostasis. Here, we identified a novel homozygous variant of *KCNJ16*, *I26T*, in an Amish patient affected with polydipsia, developmental delay, and chronic metabolic acidosis with low serum bicarbonate concentration. Subsequently, we generated the rat model with *I26T* mutation using Dahl salt‐sensitive rat (I26T rat) to characterize this variant. The male mutant rats displayed similar blood pressure and electrolyte homeostasis under baseline and with a high salt (4% NaCl) challenge. Blood pH, HCO_3_^−^ and renal damage also remained similar between WT and I26T rats after high salt challenge. Additionally, single‐channel patch clamp analysis revealed similar channel activity in CHO cells overexpressed with WT and *I26T* mutant K_ir_4.1/5.1 channels. In summary, this study reported a novel variant in *KCNJ16*, namely *I26T*, which is likely a benign variant and not associated with pathologic phenotype in either human or Dahl salt‐sensitive rats, indicating that the type/location of variant should be considered when diagnosing and treating patients with *KCNJ16* mutations.

## 1 INTRODUCTION

In the kidney, Na^+^ and K^+^ transport in the aldosterone‐sensitive distal nephron (ASDN) is essential for determining the pressure‐natriuresis relationship responsible for the long‐term control of arterial blood pressure (Staruschenko, 2012). As the predominant basolateral K^+^ channel in the ASDN, the heterotetramer of inwardly rectifying K^+^ channels K_ir_4.1 (*KCNJ10*) and K_ir_5.1 (*KCNJ16*), namely, K_ir_4.1/5.1, plays an important role in controlling resting membrane potential and maintaining the Na^+^/K^+^‐ATPase activity (Manis et al., 2019; Palygin et al., 2017). In humans, loss‐of‐function mutation of *KCNJ10* has been suggested to cause EAST/SeSAME syndrome, which is characterized as renal salt wasting, hypokalemia alkalosis, epilepsy, ataxia, and sensorineural deafness (Mir et al., 2019; Scholl et al., 2009). More recent case reports demonstrated that different mutations of *KCNJ10* are associated with other symptoms in humans, For example, a case series of 4 adult patients combined with *in‐silico* modeling indicated that truncating mutations of *KCNJ10* are associated with more severe disease (Nadella et al., 2019; Suzumoto et al., 2021). Similarly, defects of *KCNJ16* in humans cause hypokalemia, salt wasting, disturbed acid–base homeostasis, and sensorineural deafness (Chen et al., 2023; Schlingmann et al., 2021; Webb et al., 2021). Here, we identified a new variant of *KCNJ16* in patients and characterized it in the Dahl salt‐sensitive (SS) rat model and mammalian cells overexpressing the channel subunits.

## 2 MATERIALS AND METHODS

### 2.1 Patient data acquisition and whole exome sequencing

The study was approved by DDC Clinic for Special Needs Children Institutional Review Board (#IRB00006147), conformed to the Declaration of Helsinki, and written informed consent was obtained from each participant or their legal guardian. Subjects were clinically evaluated by physicians at DDC Clinic, and additional clinical information was collected from other medical facilities. The whole exome sequencing was performed on all subjects using Illumina platforms. Sanger sequencing was performed for variant confirmation.

### 2.2 Generation of I26T mutant rats

*I26T* gene variant in *Kcnj16* on the genetic background of the Dahl SS rat was produced by injecting a CRISPR‐Cas9 targeting the *Kcnj16* exon 1 sequence TCGGCTATGGCATGCTCTGG into the embryos of SS/JrHsdMcwi rats along with a single‐strand oligonucleotide of sequence GCTGCCATCTTTGTGGAGCAGGCGCCTCCTCGCTCTTCTCTTCTCGGCT**g**TGGCgTGtTCTGGAGGATAGCCTGGATATTTGGAGTCCACGTTGACAATCCGGTAGCTACTTCCGTAATA to incorporate the isoleucine to threonine (I26T) missense substitution (bold lowercase) plus two additional silent mutations (lowercase) to prevent Cas9 cleavage after homology‐directed repair. The resulting variants cause 3 base pair substitutions in the following region of exon 1: (mRatBN7.2/rn7): chr10:99,388,240‐99,388,248, causing the single amino acid substitution (I to T). Founder animals were genotyped by Sanger sequencing, then backcrossed to the parental strain, and an SS‐Kcnj16^em9Mcwi^ (SS^*I26T+/−*^) (RGDID:207424620) breeding colony was established. In this study, male homozygous (I26T) and wild‐type (WT) littermates were used.

### 2.3 Dietary protocol and animal handling

All animal experiments were conducted in accordance with the National Institute of Health Guide for the Care and Use of Laboratory Animals, following protocols reviewed and approved by the University of South Florida Institutional Animal Care and Use Committee. Rats were housed in controlled environmental conditions under a 12‐h‐light/dark cycle with 0.4% NaCl diet (Dyets Inc., D113755) and water provided ad libitum as described (Zietara et al., 2023).

### 2.4 Blood pressure and urine/serum biochemistry

At the age of 8–9 weeks, a blood pressure transmitter (PA‐C40; DSI) was surgically implanted subcutaneously into the rats with the catheter tip secured in the abdominal aorta via the femoral artery. After a 5–7 days recovery period, the rats were placed into metabolic cages for 24‐h urine collection. The rats were then switched to a 4% NaCl diet (Dyets Inc., D113756) for 3 weeks with blood pressure and heart rate measurement in conscious, freely moving WT and I26T rats using a DSI system. 24‐h urine was collected every 7 days. At the completion of the study, rats were anesthetized with 2%–3% (vol/vol) isoflurane and surgically prepared for a kidney flush and arterial blood collection. Blood pH, pCO_2_ and blood and urine electrolytes were analyzed using the ABL800 FLEX blood gas analyzer (Dissanayake et al., 2022; Palygin et al., 2017). Blood HCO_3_^−^ concentration was calculated from the measurement of pH and pCO_2_ using the Henderson‐Hasselbach equation (Hennings et al., 2012).

### 2.5 Histological analysis

The right kidneys were harvested for histological analysis as previously described (Xu et al., 2022). Briefly, the harvested kidneys were fixed by 10% formalin and then embedded by paraffin. The kidney sections were cut at 4 μm thickness and then stained with Masson's trichrome. Protein cast and fibrosis analyses were performed with a color thresholding method using Qupath (v.0.4.2) (Bankhead et al., 2017).

### 2.6 Electrophysiology

Chinese Hamster Ovary (CHO) (RRID:CVCL_0213) cells were obtained from American Type Culture Collection (ATCC; Manassas, VA, USA) and maintained under standard culture conditions (F12K medium supplemented with 10% FBS, 10 U/mL penicillin and 100 μg/mL streptomycin) as previously described (Ilatovskaya et al., 2011; Staruschenko et al., 2005). Cells were trypsinized, plated into 35‐mm tissue culture dishes with coverslips, and grown for 20–24 h prior to transfection with PolyFect Transfection Reagent (QIAGEN; Germantown, MD, USA). For overexpression of WT and *I26T* mutant K_ir_4.1/5.1 channels in CHO cells, the cells were transiently co‐transfected with eGFP (Addgene; Watertown, MA, USA) (0.25 μg of cDNA/35‐mm dish) and channel plasmids. A WT bicistronic vector encoding the K_ir_4.1/5.1 channel (pBUDCE4.1‐human *KCNJ10/KCNJ16*) was kindly provided by Dr. Jerod S. Denton (McClenahan et al., 2022). A mutant variant of the channel was generated by introducing a point mutation at the isoleucine codon to a threonine codon (Ile26Thr) in the *KCNJ16* gene. The mutant construct (217B pBUDCE4.1 hKCNJ10 hKCNJ16‐Ile‐26‐Thr) was synthesized by Custom DNA Construct (Islandia). After transfection, cells were cultured on coverslips for 24–48 h, and GFP‐positive cells were selected for measurements.

The single‐channel activity was determined in cell‐attached patches mode under voltage‐clamp configuration as previously described (Isaeva et al., 2022). Data were acquired and subsequently analyzed with Axopatch 200B amplifier interfaced via a Digidata 1440A to a PC running the pClamp 10.6 suite of software (Molecular Devices; RRID:SCR_ 011323). The recordings were filtered with an 8‐pole, low‐pass Bessel filter LPF‐8 (Warner Inst.) at 0.3. Events were inspected visually before acceptance to ensure that patches contained activity consistent with the channel of interest and were not contaminated with other conductance. Automatically detected event histograms fit with single or multiple Gaussian distributions were used to determine unitary current amplitudes (Clampfit 10.3 software). All electrophysiological experiments were performed at room temperature (22–24°C). The patch pipettes were pulled with a horizontal puller (Sutter P‐97; Sutter Inst.), and their resistances ranged from 7 to 12 MΩ. All electrophysiological recordings were performed using PSS as an extracellular solution (in mM): 150 NaCl, 5 KCl, 1 CaCl_2_, 2 MgCl_2_, 5 glucose and 10 HEPES (pH 7.35). The single‐channel activity of K_ir_4.1/5.1 and K_ir_4.1 channels was measured using patch pipettes filled with a solution of the following composition (in mM): 150 KCl, 2 MgCl_2_ and 10 HEPES (pH 7.35).

### 2.7 Statistical analysis

Data are expressed as mean ± SD and analyzed by GraphPad Prism 10 (GraphPad Software, San Diego, CA) and compared by the Student's *t*‐test. *p < 0.05, **p < 0.01.

## 3 RESULTS

### 3.1 Clinical case description

The patient was a 2‐year‐old Amish male presented at DDC Clinic with polydipsia, developmental delays, and failure to thrive. Other clinical manifestations included hypotonia, constipation, and mild chronic metabolic acidosis with low serum bicarbonate concentration (15 to 20 mmol/L); thus inherited renal tubulopathy was suspected. Although his serum electrolytes and blood pressure were in the normal ranges during the clinical visits at DDC Clinic, his clinical symptoms partially responded to empirical therapy of potassium bicarbonate, with significantly improved growth and development.

### 3.2 Genetic analysis

Whole exome sequencing analysis performed on DNA samples from the patient and his parents revealed a homozygous c.77T>C (p.I26T) variant in the *KCNJ16* gene in the patient and carrier status for both parents. The variant was further confirmed by Sanger sequencing. Targeted analysis of the I26T variant by Sanger sequencing showed the unaffected siblings were either carriers or normal for the variant. This variant has not been reported in the literature in association with *KCNJ16*‐related disorder, nor was it present in the ClinVar database. It was observed at an allele frequency of 0.003% (9/282778 alleles) in control populations in the gnomAD database, all in a heterozygous state. Amino acid sequence comparison showed the isoleucine residue at 26 (I26) is not conserved across species. In silico analysis predicted that this variant is likely to be tolerated to the protein function (Polyphen: benign, score 0.00; SIFT: tolerated, score 0.47).

### 3.3 Mutation of *Kcnj16* in dahl SS rats

**FIGURE 1:**
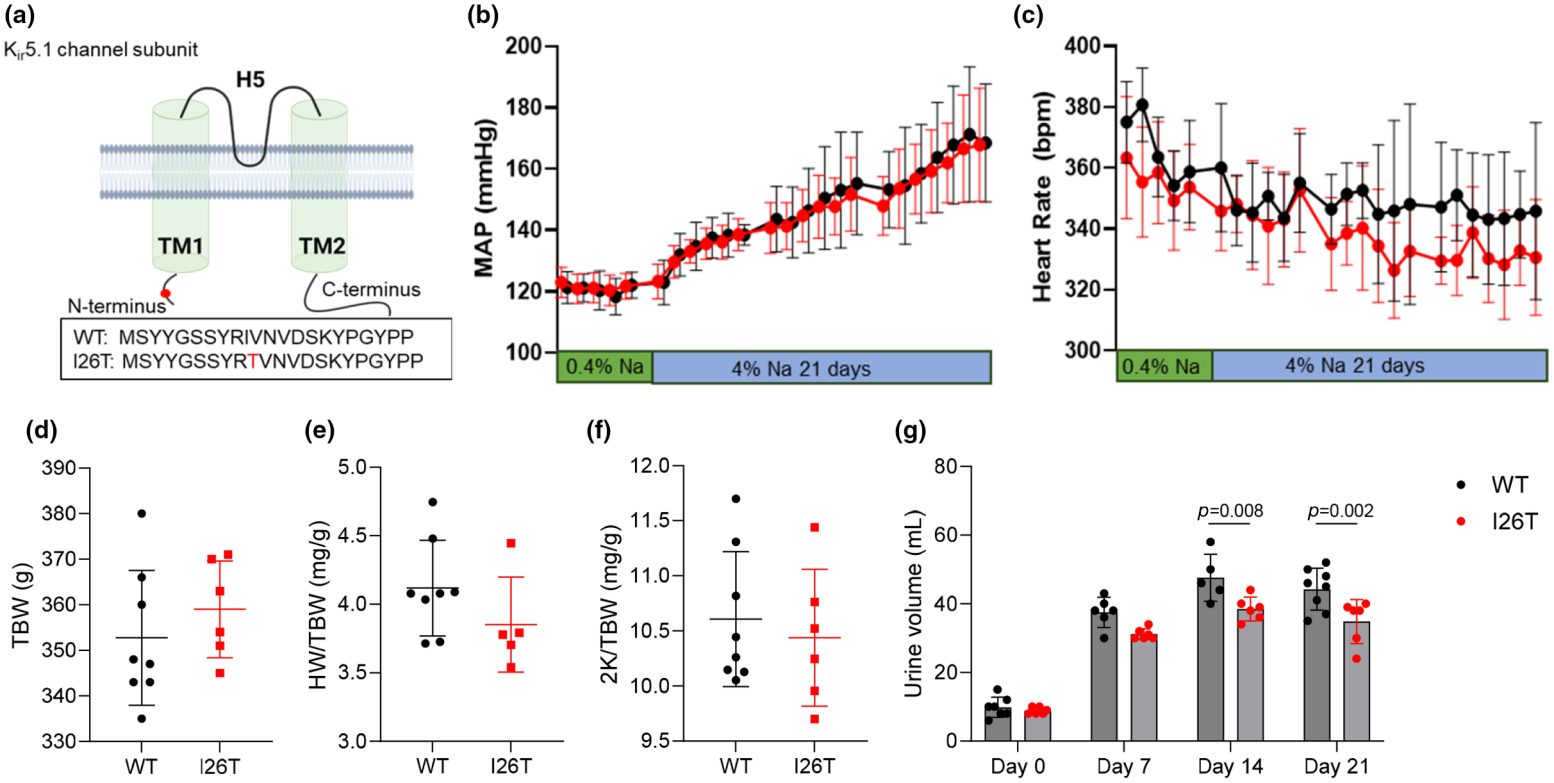
Blood pressure and physiological parameters of WT and I26T rats. (a) Substitution mutation of the *N*‐terminus of K_ir_5.1 channel in I26T rats. (b) and (c): Mean arterial blood pressure (MAP) (b) and heart rate (c) of WT and I26T rats under 0.4% NaCl and following 4% NaCl diet. (d–f): Total body weight (TBW) (d), heart‐to‐body weight ratio (HW/TBW) (e) and kidneys‐to‐body weight ratio (2 K/TBW) (f) of WT and I26T rats. (g) 24‐h urine volume of WT and I26T rats at normal salt diet (Day 0) and 7, 14, and 21 days after high slat diet. Data are expressed as mean ± SD. Different groups were compared using the Student's *t*‐test.

The Dahl salt‐sensitive (SS) rat model recapitulates the many phenotypic features found in hypertensive African Americans, making it helpful model for studying SS hypertension. To examine the role of the *KCNJ16‐I26T* variant in the development of SS hypertension as well as electrolyte homeostasis and kidney injury, we created the *Kcnj16‐I26T* mutation rats based on the Dahl SS rat background using a CRISPR‐based approach as previously described (Kitagawa et al., 2020), resulting in one amino acid substitution (I to T) at the *N*‐terminus of the protein (Figure 1a).

### 3.4 Effect of *I26T* mutation on blood pressure, electrolyte homeostasis, and kidney injury in dahl SS rats

There were no differences in the blood pressure and heart rate of WT and I26T rats at either baseline (0.4% NaCl diet) or after high salt (4% NaCl) challenge (Figure 1b,c)Total body weight, kidney weight and heart weight were also similar between WT and I26T rats after the high salt challenge (Figure 1d–f).

**FIGURE 2:**
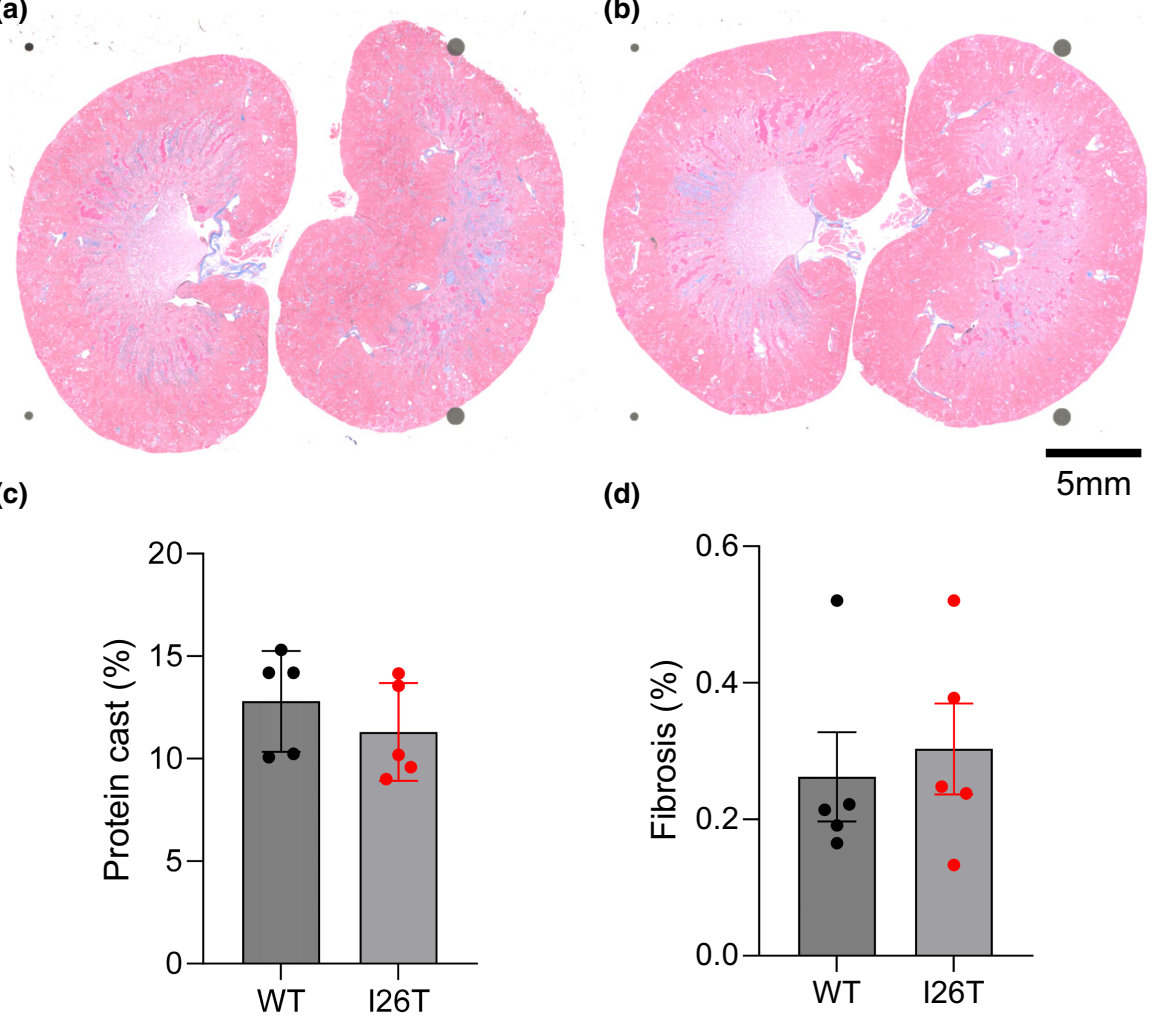
Protein cast and fibrosis analysis of the kidney of WT and I26T rats. (A and B) Representative Masson's trichrome staining of kidney tissue of WT (a) and I26T (b) rats. (c and d) Quantification of medullary protein cast (c) and cortex fibrosis (d) in kidney tissue of WT and I26T rats. Data are expressed as mean ± SD. Different groups were compared using the Student's *t*‐test.

Although there was a decrease in diuresis on days 14 and 21 in I26T rats (Figure 1g), blood and urine (Table 1) electrolytes (K^+^, Na^+^, Cl^−^, Ca^2+^) and creatinine levels at both baseline (day 0) and after high salt (4% NaCl) challenge (day 21) were similar between WT and I26T rats. Simiarly, no differences in blood pH, pCO_2_ and HCO_3_^−^ were observed between WT and I26T rats after high salt challenge (Table 1). Additionally, quantification of protein cast and fibrosis based on the Masson's Trichrome also showed no differences between WT and I26T rats (Figure 2).

**TABLE 1:** Blood and urine electrolytes of WT and I26T rats.

Blood electrolytes	WT (*N* = 8)	I26T (*N* = 6)
Blood pH	7.31 ± 0.05	7.34 ± 0.03
Blood pCO_2_	56.2 ± 8.2	52.6 ± 4.5
Blood HCO_3_^−^	26.9 ± 1.2	27.4 ± 1.0
K^+^, mM	3.5 ± 0.2	3.6 ± 0.2
Na^+^, mM	139 ± 2	139 ± 1
Ca^2+^, mM	1.34 ± 0.03	1.33 ± 0.04
Cl^−^, mM	103 ± 3	102 ± 4
Creatinine, mg/dL	0.46 ± 0.12	0.35 ± 0.09

Urine electrolytes	Day 0		Day 21	
	WT (*N* = 7)	I26T (*N* = 6)	WT (*N* = 8)	I26T (*N* = 6)
K^+^/Cre	15.5 ± 5.4	13.2 ± 2.4	12.5 ± 1.2	13.3 ± 1.5
Na^+^/Cre	17 ± 5	15 ± 5	102 ± 46	84 ± 16
Ca^2+^/Cre	0.11 ± 0.05	0.12 ± 0.04	0.76 ± 0.43	1.08 ± 1.23
Cl^−^/Cre	88 ± 19	34 ± 8	102 ± 12	129 ± 38
Creatinine, mM	7.4 ± 2.7	6.9 ± 2.3	3.4 ± 1.4	3.5 ± 1.5

*Note*: Data are expressed as mean ± SD.

### 3.5 K_ir_4.1/5.1 channel activity

**FIGURE 3:**
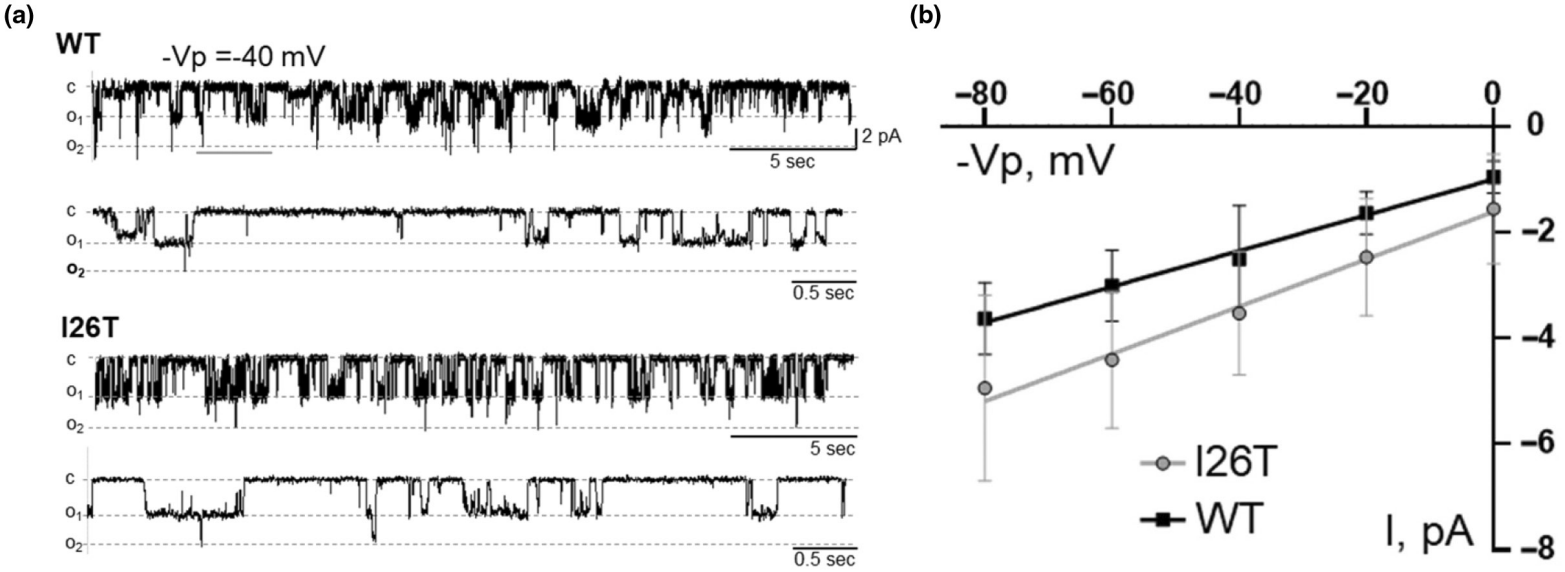
Functional properties of WT and *I26T* mutant K_ir_4.1/5.1 channels overexpressed in Chinese Hamster Ovary (CHO) cells. (a) Representative traces of single‐channel recordings of WT and *I26T* mutant K_ir_4.1/5.1 channels at a holding potential ‐Vp = 40 mV. For both genotypes, the top panel indicates a 5‐s scale segment of the recordings, and the bottom panel indicates an increased 0.5‐s scale segment of the same trace, providing a detailed view of the channel opening and closing events. Each trace represents the channel opening (o) and closing (c) events observed during the recordings, demonstrating the similar functional properties of the WT and mutant channels. (b) Current (I)‐voltage (−Vp) relationship of the WT and I26T mutant K_ir_4.1/5.1 channels.

Consistent with our phenotypic animal studies, single‐channel patch clamp analysis on cultured CHO cells over‐expressed with WT and *I26T* mutant K_ir_4.1/5.1 channels revealed no difference in K_ir_4.1/5.1 channel activity (Figure 3).

## 4 DISCUSSION

Several studies have reported mutations in *KCNJ16* in patients. For example, *Schingmann* et al. identified 6 missense mutations in 8 patients from 7 families. These patients included both males and females, ranged from 14 months to 22 years old and displayed hypokalemia, salt wasting, disturbed acid‐bae homeostasis, and sensorineural deafness (Schlingmann et al., 2021). Another study by *Webb* et al. reported a 2‐year‐old female with biallelic loss‐of‐function variants in *KCNJ16*, presenting with hypokalemia and chronic metabolic acidosis with exacerbations triggered by minor infections (Webb et al., 2021). More recently, a novel *KCNJ16* variant was identified in a Chinese patient with hypokalemic metabolic acidosis (Chen et al., 2023).

Here, we identified a novel variant of *KCNJ16*, namely, *I26T*, which causes one amino acid substitution (I to T) at the *N*‐terminus of the protein. Interestingly, although the original patient presented with various clinical manifestations such as hypotonic and mild metabolic acidosis, this is likely not due to the *I26T* variant, as further sequencing analysis of 1426 healthy Amish control samples (*N*=2852 chromosomes) revealed an allele frequency of 4.3% (123/2852 alleles), including four homozygous individuals, indicating that it is more likely a population‐specific benign variant. Consistently, Dahl SS rats with the same mutation also have similar blood pressure and electrolyte homeostasis both under baseline (0.4% NaCl diet) and high salt challenge (4% NaCl diet) as WT rats (Figure 1 and Table 1). Blood pH, HCO_3_^−^ and the kidney injury after high salt challenge also remains the same between WT and I26T rats (Figure 2 and Table 1). These observations are further confirmed by the similar K_ir_4.1/5.1 channel activity in CHO cells overexpressed with WT and *I26T* mutant K_ir_4.1/5.1 channel subunits (Figure 3). Although there is a slight decrease of diuresis at day 14 and 21 of high salt challenge, the effect is very mild and does not have any impact on blood pressure or electrolytes (Figure 1g).

After high salt, the phenotype of the SS rats with *I26T* mutation is dramatically different from the SS rats lacking K_ir_5.1 (SS^Kcnj16−/−^), where SS^Kcnj16−/−^ rats present low blood pressure, severe hypokalemia, and salt wasting phenotype (Palygin et al., 2017). The profound differences between the clinical presentation of the newly identified and characterized *I26T* variant in *KCNJ16* and the previously reported mutations might be due to the different mutation type/location, which largely affect the function and activity of the channel. The influences of mutation types on clinical presentations in patients have been demonstrated in different diseases (Mehyar et al., 2020; Suzumoto et al., 2021). In the case of *KCNJ10*, the prognosis of patients suffering from EAST/SeSAME syndrome is related to the severity of the mutation causing the disease, with the patients with truncating mutations of *KCNJ10* associated with more severe outcomes both in tubulopathy severity and neurological symptomology (Suzumoto et al., 2021). Similarly, the location of the mutation may also play a role. For example, 70% of patients with Rett's syndrome, a pervasive neurological disorder characterized by symptoms like compromised brain functions, and severe mental retardation, have mutations of the *MECP2* gene at specific CpG hot spots in exons 3 and 4 (Liyanage & Rastegar, 2014; Petel‐Galil et al., 2006). Compared to *KCNJ16‐I26T* variant, the location of other reported pathogenic *KCNJ16* variants include missense mutations at the pore‐forming domain near the selectivity filter of the channel, N‐terminus near the first transmembrane domain and *C*‐terminus (Chen et al., 2023; Schlingmann et al., 2021; Webb et al., 2021). Additionally, 22.2% of the pathogenic *KCNJ16* variants are nonsense variants, while 77.8% are missense variants (Chen et al., 2023).

In summary, this study identified and characterized a novel variant of *KCNJ16*, namely *I26T*, which is likely a benign variant and not associated with pathologic phenotype in either human or Dahl salt‐sensitive rats, indicating that the type/location of the variant should be considered when diagnosing and treating patients with *KCNJ16* mutations.

## AUTHOR CONTRIBUTIONS

A.S. and H.W. conceptualized the study. A.M.G. generated the I26 mutant animal model. B.X. performed and analyzed the data from animal experiments. V.L. performed telemetry surgery and organ extraction. R.B. and A.A. performed and analyzed the data from electrophysiology experiments. V.S. and B.Z.X. performed human studies. B.X. wrote the original draft. B.X., V.L., R.B., A.A., A.M.G., B.Z.X., H.W. and A.S. reviewed and edited the manuscript. All authors approved the final version.

## FUNDING INFORMATION

This work was supported by National Institutes of Health Grants R35 HL135749 (to A.S.) and the Vascular Inflammation and Injury Training Program T32 HL160529 (to R.B.) and Department of Veteran Affairs grants I01 BX004024 (to A.S.). The contents of this manuscript do not represent the views of the Department of Veterans Affairs or the United States Government.

## CONFLICT OF INTEREST STATEMENT

The authors declare no conflicts of interest.

## ETHICS STATEMENT

Patient studies were approved by DDC Clinic for Special Needs Children Institutional Review Board. All animal experiments were conducted in accordance with the National Institute of Health Guide for the Care and Use of Laboratory Animals, following protocols reviewed and approved by the University of South Florida Institutional Animal Care and Use Committee.

## Data Availability

The data that support the findings of this study are available from the corresponding author upon reasonable request.
